# CYP1A2 expression rather than genotype is associated with olanzapine concentration in psychiatric patients

**DOI:** 10.1038/s41598-023-45752-6

**Published:** 2023-10-28

**Authors:** Ferenc Fekete, Ádám Menus, Katalin Tóth, Ádám Ferenc Kiss, Annamária Minus, Dávid Sirok, Aleš Belič, Ádám Póti, Gábor Csukly, Katalin Monostory

**Affiliations:** 1 Institute of Enzymology, HUN-REN Research Centre for Natural Sciences, Magyar tudósok 2, Budapest, 1117 Hungary; 2 Doctoral School of Biology and Institute of Biology, Eötvös Loránd University, Pázmány Péter Sétány 1/A, Budapest, 1117 Hungary; 3 Department of Psychiatry and Psychotherapy, Semmelweis University, Balassa 6, Budapest, 1082 Hungary; 4 Toxi-Coop Toxicological Research Center, Magyar jakobinusok 4/B, Budapest, 1122 Hungary; 5 Lek Pharmaceuticals d.d., Kolodvorska 27, 1234 Menges, Slovenia

**Keywords:** Genetics research, Molecular medicine

## Abstract

Olanzapine is a commonly prescribed atypical antipsychotic agent for treatment of patients with schizophrenia and bipolar disorders. Previous in vitro studies using human liver microsomes identified CYP1A2 and CYP2D6 enzymes being responsible for CYP-mediated metabolism of olanzapine. The present work focused on the impact of *CYP1A2* and *CYP2D6* genetic polymorphisms as well as of CYP1A2 metabolizing capacity influenced by non-genetic factors (sex, age, smoking) on olanzapine blood concentration in patients with psychiatric disorders (N = 139). *CYP2D6* genotype-based phenotype appeared to have negligible contribution to olanzapine metabolism, whereas a dominant role of CYP1A2 in olanzapine exposure was confirmed. However, CYP1A2 expression rather than *CYP1A2* genetic variability was demonstrated to be associated with olanzapine concentration in patients. Significant contribution of − 163C > A (rs762551), the most common SNP (single nucleotide polymorphism) in *CYP1A2* gene, to enhanced inducibility was confirmed by an increase in CYP1A2 mRNA expression in smokers carrying − 163A, and smoking was found to have appreciable impact on olanzapine concentration normalized by the dose/bodyweight. Furthermore, patients’ olanzapine exposure was in strong association with CYP1A2 expression; therefore, assaying CYP1A2 mRNA level in leukocytes can be an appropriate tool for the estimation of patients’ olanzapine metabolizing capacity and may be relevant in optimizing olanzapine dosage.

## Introduction

Schizophrenia is a chronic mental disorder with a relatively low prevalence (less than 1%), however, with severe symptoms, such as hallucinations, delusions, cognitive impairment and distortion of thinking and behaviour^1^. The mainstay of its pharmacotherapy is based on antipsychotic drugs that efficiently reduce psychotic symptoms. Olanzapine, one of the most widely prescribed second generation (atypical) antipsychotics, is efficient against both positive and negative symptoms of schizophrenia; furthermore, it is also effective in treatment of manic or mixed episodes of bipolar disorder^2–4^. Olanzapine is a potent antagonist of various dopamine (D_1_, D_2_, D_3_, D_4_) and serotonin (5-HT_2A_, 5-HT_2B_, 5-HT_2C_, 5-HT_3_) receptor subtypes, and also exhibits affinity to muscarinic, α_1_-adrenergic and histamine H_1_ receptors^5^. Although the mechanism of antipsychotics action in monoamine neurotransmission system is complex, lower selectivity of olanzapine for D_2_ receptor is considered to be associated with more favorable adverse effect profile and less prevalent extrapyramidal symptoms (such as akathisia, parkinsonism and tardive dyskinesia) compared to the first generation (typical)^6,7^ or other atypical antipsychotics^8,9^. The most common side effects induced by olanzapine are metabolic disturbances (e.g., weight gain, hyperglycemia, elevated serum cholesterol and triglyceride levels), hyperprolactinemia, orthostatic hypotension, sedation and anticholinergic adverse effects (constipation, dry mouth)^10^.

The inappropriate dosage can lead to the lack of improvement of symptoms or development of severe adverse effects, and eventually to discontinuation of olanzapine therapy^11,12^. Olanzapine plasma concentrations have been reported to be associated with patients’ response to the treatment (therapeutic window of 20–80 ng/mL), and the risk of adverse effects was demonstrated to increase with plasma concentration; therefore, therapeutic drug monitoring of olanzapine is highly recommended^13–15^. Substantial interindividual variability in olanzapine pharmacokinetics has been observed which was attributed to the variations in the activities of olanzapine metabolizing enzymes. Olanzapine is extensively metabolized in the liver via glucuronidation by UDP-glucuronyltransferases (primarily by UGT1A4)^16,17^ and via oxidative metabolism by cytochrome P450 (CYP) and flavin-containing monooxygenase (FMO) enzymes. The major pathways of oxidative metabolism are catalyzed by CYP1A2 and FMO3 resulting in 4′-*N*-desmethyl-olanzapine and olanzapine *N*-oxide, respectively; however, the minor routes leading to the formation of hydroxy-metabolites (2-hydroxy- and 7-hydroxy-olanzapine) involve CYP2D6 and CYP1A2 enzymes^18,19^. These metabolites are pharmacologically less active than the parent compound, and do not contribute significantly to the therapeutic effect of olanzapine^20^.

Genetic polymorphisms of drug metabolizing enzymes have been assumed to be responsible for the interindividual variability in olanzapine pharmacokinetics; however, no clinical guideline and recommendations based on genetically determined metabolism have been prepared for olanzapine dosing. Although UGT1A4 (and UGT2B10 to a less extent) is considered to be the catalyst of one of the major routes of olanzapine metabolism, the functional impact of *UGT* polymorphisms on olanzapine exposure appears to be controversial^21–23^. In vitro enzyme mapping studies using human liver microsomes and cDNA expressed microsomal enzymes suggested that FMO3 and CYP enzymes can contribute to interindividual differences in olanzapine pharmacokinetics^19^. Genetic polymorphisms of *FMO3* have been demonstrated to be associated merely with olanzapine *N*-oxide formation in patients, and no significant impact was observed on olanzapine plasma concentrations^18,24^. The influence of inherited differences in CYP2D6 and CYP1A2 function on olanzapine metabolism has been extensively studied; however, clear association between olanzapine concentration and the polymorphic *CYP* alleles has hardly been demonstrated^17,18,25^.

Hepatic CYP1A2 and CYP2D6 activities display more than 100-fold interindividual variability which is partly attributed to the genetic polymorphisms of *CYP1A2* and *CYP2D6*, and non-genetic factors (age, gender, diseases, medication, smoking) evoking phenoconversion, and has significant impact on patients’ drug metabolizing capacity^26–28^. Several clinically relevant *CYP2D6* alleles have been identified, many of which are associated with decreased or even no CYP2D6 activity (CYP2D6*3, CYP2D6*4, CYP2D6*5, CYP2D6*6, CYP2D6*9, CYP2D6*10, CYP2D6*41), whereas multiplication of functional allele (×*N*) has been linked to ultrarapid metabolism of CYP2D6 substrates^27,29,30^. According to the Clinical Pharmacogenetics Implementation Consortium (CPIC), *CYP2D6* genotyping allows a fairly consistent prediction for phenotypes referred to poor (PM), intermediate (IM), normal (NM) and ultrarapid metabolizers (UM); however, the limitations of pharmacogenetic based estimation of drug metabolizing phenotypes must be considered^31,32^. Concomitant use of CYP2D6 inhibitor drugs (e.g., fluoxetine, paroxetine, quinidine, bupropion) or non-CYP2D6-specific influences (e.g., chronic alcohol consumption, comedication with non-CYP2D6 inhibitor drugs causing liver disfunction) have been reported to modify the genotype-based phenotype, resulting in genotype–phenotype mismatch; therefore, composite phenotype is suggested to be applied in personalized dosing of a CYP2D6-substrate drug^28,33,34^.

For CYP1A2, the identification of single nucleotide polymorphisms (SNPs) in *CYP1A2* gene provides a relatively weak prediction for CYP1A2 phenotype^35^. Some of the *CYP1A2* SNPs, such as − 3860G > A (rs2069514), − 2467delT (rs35694136), − 739T > G (rs2069526), − 163C > A (rs762551) and 2159G > A (rs2472304), are present in several *CYP1A2* haplotypes, which makes the identification of allelic variants laborious; furthermore, the functional relevance of a particular SNP to CYP1A2 activity may differ from that of the haplotype^35,36^. Decreased CYP1A2 activity has been reported to be associated with the nucleotide change of − 3860G > A and − 2467delT^37,38^, whereas the SNP at the position − 163C > A has been suggested to be associated with high inducibility of *CYP1A2* transcription in smokers; however, the effect of − 163C > A SNP itself on CYP1A2 activity is highly inconsistent^37,39,40^. Furthermore, the phenoconverting non-genetic factors (smoking, age, sex, hormones, diseases, medication) can mask the effect of *CYP1A2* allelic variants resulting in transient poor or extensive drug metabolizing activity^39,41,42^.

The main aim of the present study was to evaluate the contribution of *CYP1A2* and *CYP2D6* genetic variability as well as of CYP1A2 expression to olanzapine exposure in patients with schizophrenia and bipolar disorders. To avoid misinterpretation of *CYP1A2* genetic polymorphisms and its role in olanzapine pharmacokinetics, we included the SNPs most prevalent in Caucasian populations in the evaluation. The further aim of the present study was to analyze the influence of phenoconverting non-genetic factors (age, sex, smoking behaviour) on olanzapine metabolizing capacity of the patients.

## Materials and methods

### Patients

Inpatients (N = 139) treated with schizophrenia, schizoaffective or bipolar disorders at the Department of Psychiatry and Psychotherapy, Semmelweis University (Budapest, Hungary) were enrolled in the present study. Inclusion criteria were the age of 18 years or older and stable olanzapine therapy for at least two weeks. Exclusion criteria were drug or alcohol addiction. The study was approved by the Hungarian Committee of Science and Ethics, Medical Research Council. It was performed under the regulations of Act CLIV of 1997 on Health and the decree 23/2002 of the Minister of Health of Hungary, and in accordance with the declaration of Helsinki. All patients belonged to the Caucasian population, and their demographic and clinical data as well as the details of olanzapine therapy (daily dose, serum concentration of olanzapine) were recorded (Table 1).

**Table 1:** Patients’ demographic characteristics and clinical history.

Parameter	N
Patients	139
Sex (male/female)	60/79
Age (years)^a^	39 (18; 72)
Bodyweight (kg)^a^	73 (43; 122)
Current smokers	59
Primary disease	
Schizophrenia	77
Schizoaffective disorder	25
Bipolar disorder	37
Olanzapine daily dose (mg)^a^	15 (2.5; 20)
Olanzapine serum levels (ng/mL)^a^	36.2 (1.3–170.0)

^a^Median (min; max).

Olanzapine therapy was applied according to the conventional clinical protocol with a daily dose of 2.5–20 mg. The patients’ therapy also included drugs other than olanzapine, such as antipsychotics (e.g., amisulpride, aripiprazole, cariprazine, haloperidol, risperidone, zuclopenthixol), anticonvulsants/mood stabilizers (e.g., clonazepam, lamotrigine, lithium, valproic acid), antidepressants (e.g., clomipramine, duloxetine, escitalopram, fluoxetine, mirtazapine, moclobemide, sertraline, trazodone, venlafaxine), anxiolytic drugs (e.g., alprazolam, diazepam, zolpidem, zopiclone), antibiotics (e.g., amoxicillin + clavulanic acid, clarithromycin, ciprofloxacin), non-steroidal anti-inflammatory drugs (e.g., acetaminophen, acetylsalicylic acid, diclofenac, ibuprofen, metamizole), beta-adrenergic receptor blockers (e.g., bisoprolol, carvedilol, metoprolol, nebivolol, propranolol), and other anti-hypertensive agents (e.g., amlodipine, enalapril, indapamide, perindopril, ramipril, rilmenidine, valsartan).

### Assaying CYP-status

Patients’ CYP-status was determined by *CYP1A2* and *CYP2D6* genotyping and by analysing CYP1A2 expression in leukocytes. Genomic DNA was isolated from peripheral blood samples by Quick-DNA™ Universal Kit (Zymo Research, Irvine, CA). Hydrolysis SNP analyses were performed by TaqMan assays for *CYP1A2* SNPs [− 3860G > A (rs2069514), − 2467delT (rs35694136), − 739T > G (rs2069526), − 163C > A (rs762551) and 2159G > A (rs2472304)] and for *CYP2D6* SNPs [2549delA (rs35742686), 1846G > A (rs3892097), 1707delT (rs5030655), 2615-2617delAAG (rs5030656), 100C > T (rs1065852) and 2988G > A (rs28371725)]^34,35^. *CYP2D6* gene deletion and *CYP2D6* allele specific duplication/multiplication were determined as previously described^34^.

For assaying CYP1A2 mRNA expression, total RNA was extracted from leukocytes (approximately 10^7^ cells) isolated from peripheral blood samples using TRIzol reagent (Invitrogen, Carlsbad, CA), according to the manufacturer’s instructions. RNA (3 µg) was reverse-transcribed into single-stranded cDNA using Maxima First Strand cDNA Synthesis Kit (Thermo Fisher Scientific, Waltham, CA), and real-time PCR (polymerase chain reaction) with human cDNA was performed using KAPA Fast Probes Mastermix (KAPA Biosystems, Cape Town, South Africa) and TaqMan probe for CYP1A2 (Microsynth AG, Balgach, Switzerland). The quantities of the CYP1A2 mRNA relative to that of the housekeeping gene glyceraldehyde 3-phosphate dehydrogenase (*GAPDH*) were determined^43^. Patients were classified as low, normal and high CYP1A2 expressers on the basis of CYP1A2 mRNA levels in leukocytes with the cut-off expression values of 10^−5^ and 5 * 10^−4^ as previously determined^35^.

### Plasma concentrations of olanzapine

The blood samples were taken directly before the morning dose of olanzapine, 12 h after the evening dose. The blood samples were taken at the same time for CYPtesting and for therapeutic drug monitoring. Olanzapine plasma concentration was determined by LC–MS/MS using an Inertsil ODS-4 column (75 × 2.1 mm, 3 µm; GL Sciences Inc., Tokyo, Japan) and mobile phases of 0.1% formic acid and acetonitrile in gradient running mode. The samples were analyzed using positive electrospray ionization (Sciex API 2000, MDS AB Sciex, Toronto, Canada) and multiple reaction monitoring mode for quantitation of olanzapine (*m/z* 313.3/256.1 and 313.3/198.1). Plasma concentrations divided by the corresponding 24-h olanzapine dose on a mg/kg basis were calculated for normalized olanzapine plasma concentrations.

### Data analysis

*CYP1A2* haplotypes (CYP1A2*1C, CYP1A2*1D, CYP1A2*1E, CYP1A2*1F, CYP1A2*1L, CYP1A2*1M, CYP1A2*1V, CYP1A2*1W), *CYP2D6* haplotypes (CYP2D6*3, CYP2D6*4, CYP2D6*5, CYP2D6*6, CYP2D6*9, CYP2D6*10, CYP2D6*41, CYP2D6*1xN), CYP1A2 mRNA levels in leukocytes and olanzapine plasma concentrations were determined in 139 psychiatric inpatients. We used PHASE software (v2.1; Department of Statistics, University of Washington, Seattle, WA) for reconstructing the *CYP1A2* haplotypes from the SNP data^44,45^. The genetic linkage between *CYP1A2* SNPs was demonstrated on the basis of linkage disequilibrium coefficient (D′), correlation coefficient (R^2^) and logarithm of odds (LOD) calculated by the software Haploview (v. 4.2; Broad Institute, Cambridge, MA)^46^. Strong linkage disequilibrium was identified between a pair of SNPs with D′ value more than 0.99 and LOD ≥ 3.

Linear regression models were built for identification of potential associations between olanzapine plasma concentration as a dependent variable (experimental results) and the independent variables, such as *CYP1A2* and *CYP2D6* SNPs, haplotypes, CYP1A2 expression, sex, age (under 50 and over 50 years old) and smoking behaviour (part of experimental setup). Multiple linear regression analyses were carried out by IBM SPSS Statistics software [v28.0.1.0 (142), IBM Corp., Armonk, NY, USA]. A *P* value of < 0.05 was considered to be statistically significant.

Statistical significance of CYP1A2 expression, *CYP2D6* genotype and smoking behaviour as covariates of olanzapine plasma concentrations was also analyzed by principal component analysis (PCA) and partial least-square (PLS) modelling (SIMCA, Sartorius Stedim Data Analytics AB, Umea, Sweden). PCA is a linear method that is efficiently used to decorrelate variables in multivariate data analysis. The method is particularly useful when independent variables cannot be decorrelated upfront via design of experiments (often the case in clinical studies) to extract most from the experimental results and to avoid spurious correlations between independent and dependent variables. PLS provides linear regression models similar to multiple regression; however, it uses PCA to first decorrelate the independent variables of the model. PLS models are identical to multiple regression models when independent variables are uncorrelated and are less biased than multiple regression models when independent variables are correlated. All independent variables of the study were selected as PLS model input variables while predicting olanzapine plasma concentration. The final PLS model was then cleaned of all the independent variables that did not significantly contribute to the model prediction. Cleaning was done on the basis of significance and clinical relevance of each independent variable. Contribution of a variable was considered as significant when estimated corresponding coefficient’s absolute value was larger than the estimated standard deviation of the coefficient. In the selection process, centered and scaled model coefficients were used that allowed direct comparison between the coefficients as they were normalized by the corresponding variable’s means and standard deviations. Diagonal line and dispersion around it on observed *vs* model-based predictions of olanzapine plasma concentration plot shows how well the model correlates with measured concentrations. Coefficient of determination (R^2^) was used as a formal measure of model fit. It provided a measure of how well observed outcomes were predicted by the model. Additionally, coefficient of determination was calculated also for the part of data set not used to estimate the model coefficient and was denoted as Q^2^. Ideally R^2^ and Q^2^ should be similar for relevant models.

The comparison of normalized olanzapine plasma concentrations between various CYP1A2 or CYP2D6 groups was performed by Kruskal–Wallis ANOVA followed by Dunn’s multiple comparisons or Mann–Whitney test (GraphPad Instat v3.05; GraphPad Software, San Diego, CA, USA).

### Informed consent

Written informed consent was obtained from all participants.

## Results

### *CYP1A2* and *CYP2D6* genetic variability in psychiatric patients

**Figure 1:**
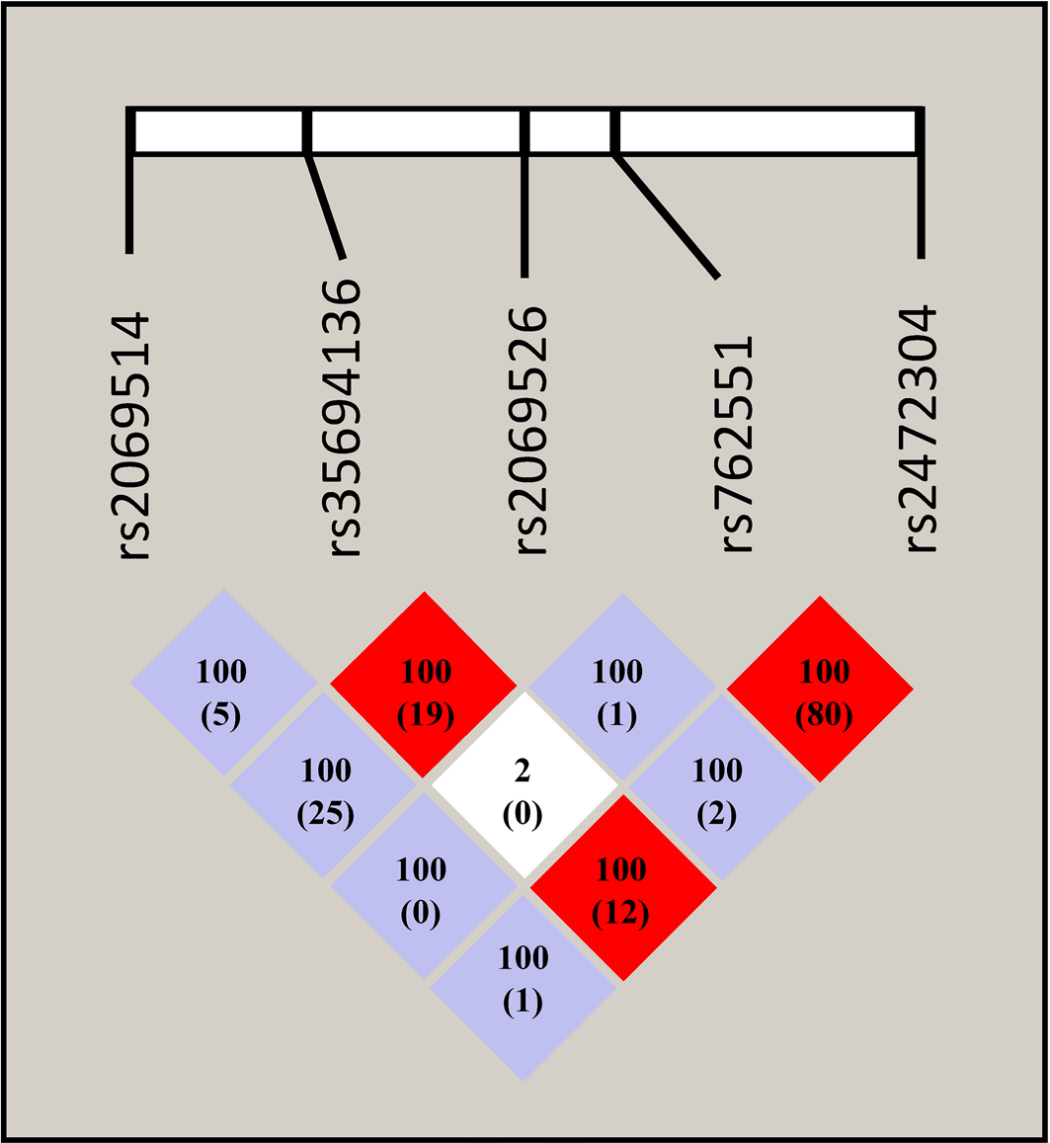
Linkage disequilibrium analysis of five SNPs in *CYP1A2*. Numbers in squares represent D' and R^2^ (in brackets) values (× 100), and the colour of each square expresses the extent of linkage disequilibrium: red, D′ = 1 LOD > 3; blue, D′ = 1 LOD < 3; white, D′ < 1 LOD < 3. The linkage disequilibrium plot was drawn using software Haploview (v4.2).

The most common *CYP1A2* (− 3860G > A, − 2467delT, − 739T > G, − 163C > A and 2159G > A) and *CYP2D6* SNPs (100C > T, 1707delT, 1846G > A, 2549delA, 2615-2617delAAG and 2988G > A) as well as *CYP2D6* gene deletion and *CYP2D6* allele specific duplication/multiplication were identified in patients with psychiatric disorders (N = 139). Subjects who did not carry any of the *CYP1A2* or *CYP2D6* polymorphisms were considered to have the CYP1A2*1 and CYP2D6*1 wild-type alleles. The most common *CYP1A2* haplotype was CYP1A2*1M with a frequency of 61%, whereas the prevalence of CYP1A2*1L, CYP1A2*1V and CYP1A2*1W alleles were less than or equal to 5%. CYP1A2*1C (− 3860G > A), CYP1A2*1D (− 2467delT) and CYP1A2*1E (− 739T > G) alleles were not detected at all in the patients. According to the haplotype analysis, the relative frequencies of CYP1A2*1L (− 3860A; − 2467delT; − 163A), CYP1A2*1M (− 163A; 2159A), CYP1A2*1V (− 2467delT; − 163A) and CYP1A2*1W (− 2467delT; − 739G; − 163A) in the patients were similar to the prevalence reported in Caucasian populations, whereas the frequency of CYP1A2*1F (carried − 163A alone) markedly differed (0.4% vs. 32–57%) (Table 2)^40,47–50^. The low prevalence of CYP1A2*1F was attributed to the strong genetic linkage of − 163C > A (rs762551) with 2159G > A (rs2472304) (D′ = 1, R^2^ = 0.803, LOD = 45.92) assigning CYP1A2*1M (Fig. 1). Further genetic linkage was identified for − 2467delT (rs35694136) with 2159G > A (rs2472304) (D′ = 1, R^2^ = 0.123, LOD = 5.46), and with − 739T > G (rs2069526) (D′ = 1, R^2^ = 0.188, LOD = 3.83); however, due the low SNP frequencies, the statistical support of genetic linkage would require an increase of subject number.

**Table 2:** *CYP1A2* and *CYP2D6* allele frequencies in psychiatric patients (N = 139) and in Caucasian populations.

CYP alleles	Nucleotide changes	N	Frequency (%)	
			Psychiatric patients	Caucasian population^1^
*CYP1A2*				
*1^2^	None	86	31.2	24.4–63.5
*1C	− 3860G > A	0	0	0.4–4
*1D	− 2467delT	0	0	3.4–11
*1E	− 739T > G	0	0	0.4–6
*1F	− 163C > A	1	0.4	32–57
*1L	− 3860G > A; − 2467delT; − 163C > A; 5347T > C	1	0.4	0.8
*1M	− 163C > A; 2159G > A	169	61.2	54.8
*1V	− 2467delT; − 163C > A	14	5.1	2.8–12.3
*1W	− 3113A > G; − 2467delT; − 739T > G; − 163C > A	5	1.8	1.2–2.1
*CYP2D6*				
*1	None	160	57.6	33–40
*3	2549delA	1	0.4	0.9–1
*4	100C > T, 1846G > A	61	21.9	15–25
*5	Gene deletion	4	1.4	3–6
*6	1707delT	2	0.7	0.1
*9	2615-2617delAAG	9	3.2	2.1
*10	100C > T	10	3.6	2
*41	2988G > A	23	8.3	9
*1xN	Multiplication	8	2.9	1–9

*CYP1A2* allele information was not available for one patient.

^1^Allele frequencies in Caucasian populations^27,40,47–53^.

^2^The CYP1A2*1 wild-type allele is often designated as CYP1A2*1A.

For *CYP2D6*, the most common allelic variants were CYP2D6*4 and CYP2D6*41 with the prevalence of 21.9% and 8.3%, respectively, whereas other *CYP2D6* alleles (CYP2D6*3, CYP2D6*5, CYP2D6*6, CYP2D6*9, CYP2D6*10) and CYP2D6*1xN occurred with the frequency of less than 4% which were in accordance with the frequencies previously reported in Caucasians (Table 2)^27,51–53^. The functional impact of *CYP2D6* genetic polymorphisms on CYP2D6 activity is well characterized identifying normal (*norm*: CYP2D6*1), reduced (*red*: CYP2D6*9, CYP2D6*10 and CYP2D6*41), loss of function (*loss*: CYP2D6*3, CYP2D6*4, CYP2D6*5 and CYP2D6*6) or gain of function (*normxN*: CYP2D6*1xN) *CYP2D6* alleles^29,54^. According to the CYP2D6 activity score system of CPIC guideline, the patients with *CYP2D6loss/loss* genotype were classified as poor (PM), with *CYP2D6loss/red*, *CYP2D6red/red* or *CYP2D6norm/loss* as intermediate (IM), with *CYP2D6norm/norm* as normal (NM) and *CYP2D6norm/normxN* as ultrarapid (UM) metabolizers^55^. Based on their *CYP2D6* genotypes, more than half of the individuals (N = 75) were considered as CYP2D6 intermediate metabolizers, whereas 49 were normal, 8 were ultrarapid and 7 were poor metabolizers.

### Patients’ genetically determined drug metabolizing capacity and olanzapine exposure

**Figure 2:**
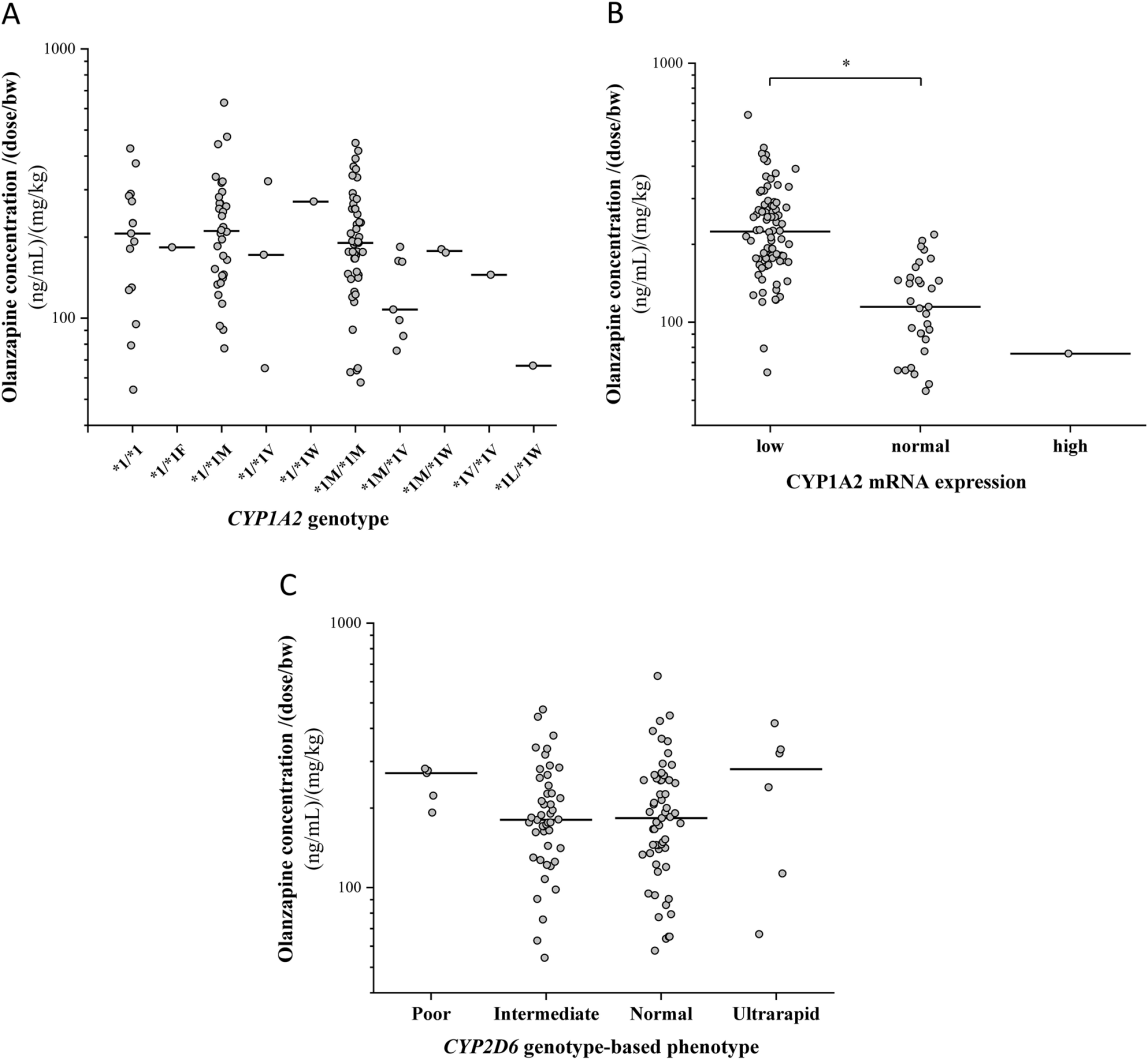
The impact of *CYP1A2* polymorphisms (**A**), CYP1A2 expression (**B**) and *CYP2D6* genotype-based phenotype (**C**) on olanzapine plasma concentrations normalized by the dose/bodyweight (bw) in patients with psychiatric disorders. *P < 0.0001.

Previous in vitro studies using human liver microsomes and cDNA-expressed microsomal enzymes have indicated that the oxidative metabolism of olanzapine is primarily CYP-mediated with a major role of CYP1A2 and with a minor role of CYP2D6^19^. The association of olanzapine plasma concentration normalized by the dose/bodyweight (C/D) with *CYP1A2* and *CYP2D6*-status was investigated in patients with psychiatric disorders. No significant differences in olanzapine plasma concentration were observed between various *CYP1A2* genotypes reconstructing from the *CYP1A2* SNP data of patients (N = 114, *P* = 0.2444) (Fig. 2A). We have previously demonstrated that CYP1A2 expression rather than *CYP1A2* genotype indicated hepatic CYP1A2 activity^35^; therefore, the association between CYP1A2 mRNA levels in the patients’ leukocytes and olanzapine concentration was also established. Due to the low number of high CYP1A2 expressers, in statistical analysis, high and normal expressers were combined. Patients with low CYP1A2 mRNA expression (N = 82) displayed approximately twofold higher olanzapine plasma concentrations than those with normal/high expression (N = 32) [239.2 ± 97.3 (ng/mL)/(mg/kg) for low versus 120.4 ± 46.8 (ng/mL)/(mg/kg) for normal/high, *P* < 0.0001] (Fig. 2B).

*CYP2D6* genotype has been suggested to be consistently applied for prediction of patients’ CYP2D6 metabolic capacity^29^; therefore, the contribution of patients’ phenotype predicted from *CYP2D6* genotype to olanzapine plasma concentration normalized by dose/bodyweight was also analyzed. No statistically significant differences in olanzapine concentration were demonstrated between poor, intermediate, normal and ultrarapid CYP2D6 metabolizer subjects [poor: 248.8 ± 39.5 (ng/mL)/(mg/kg), intermediate: 201.0 ± 91.7 (ng/mL)/(mg/kg), normal: 201.6 ± 108.6 (ng/mL)/(mg/kg), ultrarapid: 248.4 ± 136.14 (ng/mL)/(mg/kg); *P* = 0.2801] (Fig. 2C).

### Impact of genetic and non-genetic factors on olanzapine plasma concentration

**Figure 3:**
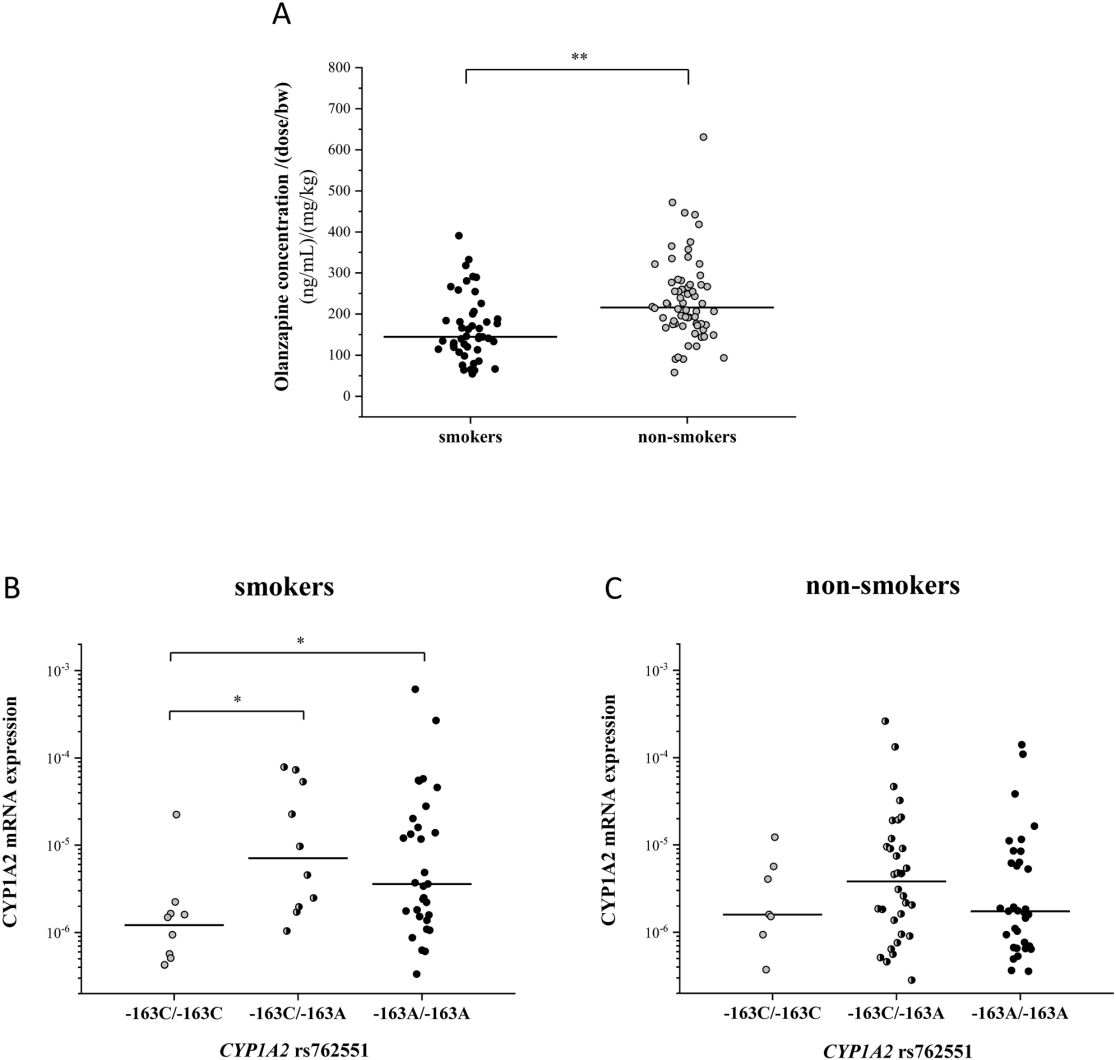
The influence of patients’ smoking behaviour on olanzapine plasma concentrations normalized by the dose/bodyweight (bw) (**A**) and on CYP1A2 expression of − 163A carrier or non-carrier patients (**B** and **C**). *P < 0.001, **P < 0.0001.

In addition to the CYP1A2 and CYP2D6 variations, a multiple linear regression analysis was performed to estimate the influence of non-genetic factors, sex and age as well as of tobacco smoking, the CYP1A2 inducing factor on olanzapine exposure in patients with psychiatric disorders (Table 3). Significant association of olanzapine plasma concentration normalized by dose/bodyweight was demonstrated both with tobacco smoking (*P* = 0.004) and with CYP1A2 expression (*P* < 0.0001). The olanzapine concentration was approximately 1.5-fold higher in non-smoker patients (N = 64) compared to smokers (N = 46) [non-smokers: 235.9 ± 102.3 (ng/mL)/(mg/kg) *vs* smokers: 165.6 ± 79.2 (ng/mL)/(mg/kg), *P* < 0.0001] (Fig. 3A). According to the multivariate analysis, none of the *CYP1A2* haplotypes had significant impact on olanzapine exposure in psychiatric patients; however, the contribution of the − 163C > A SNP was assumed to influence CYP1A2 induction by smoking. It was clearly demonstrated that smoking significantly increased CYP1A2 mRNA expression in leukocytes of patients with − 163C/A and − 163A/A nucleotide changes compared to the − 163C/C wild-type patients (C/A: 2.5*10^−5^ ± 3.1*10^−5^; A/A: 4.0 *10^−5^ ± 1.2*10^−4^ and C/C: 3.2*10^−6^ ± 6.8*10^−6^, *P* = 0.0096); however, the − 163C > A polymorphism did not have an impact on CYP1A2 transcription in non-smoker patients (Fig. 3B,C). Furthermore, the minor role of CYP2D6 in olanzapine metabolism previously demonstrated in human liver microsomes^19^ was not confirmed in patients. *CYP2D6* genotype-based phenotypes appeared to have no effect on olanzapine plasma concentration.

**Table 3:** Multivariate analysis of olanzapine plasma concentration normalized by dose/bodyweight considering genetic (*CYP1A2*, *CYP2D6* haplotypes and phenotypes) and non-genetic factors in psychiatric patients.

	Variable		Coefficient B (SE)	Coefficient β	*P* value
*CYP1A2*	Haplotypes	Constant	260.902		< 0.0001
		− 3860G/− 2467T/− 739T/− **163A**/2159G	− 72.590	− 0.070	0.406
		− 3860G/− 2467T/− 739T/− **163A**/**2159A**	− 10.010	− 0.040	0.649
		− **3860A**/− **2467delT**/− 739T/− **163A**/2159G	− 57.607	− 0.055	0.590
		− 3860G/− **2467delT**/− 739T/− **163A**/2159G	− 15.910	− 0.050	0.562
		− 3860G/− **2467delT**/− **739G**/− **163A**/2159G	− 67.154	− 0.127	0.179
	mRNA Expression	CYP1A2 Normal/high	− 99.361	− 0.448	**< 0.0001**
*CYP2D6*	Genotype-based phenotypes	Poor metabolizer	3.958	0.008	0.923
		Intermediate metabolizer	6.938	0.035	0.684
		Ultra-rapid metabolizer	71.541	0.164	0.072
Non-genetic factors		Sex	− 14.359	− 0.072	0.414
		Age	8.980	0.039	0.643
		Smoking	− 50.702	− 0.253	**0.004**

For *CYP1A2* haplotypes, the nucleotid changes are indicated in bold. The *P* values < 0.05 were considered to be statistically significant and are indicated in bold.

### Dominant factors contributing to olanzapine concentration

**Figure 4:**
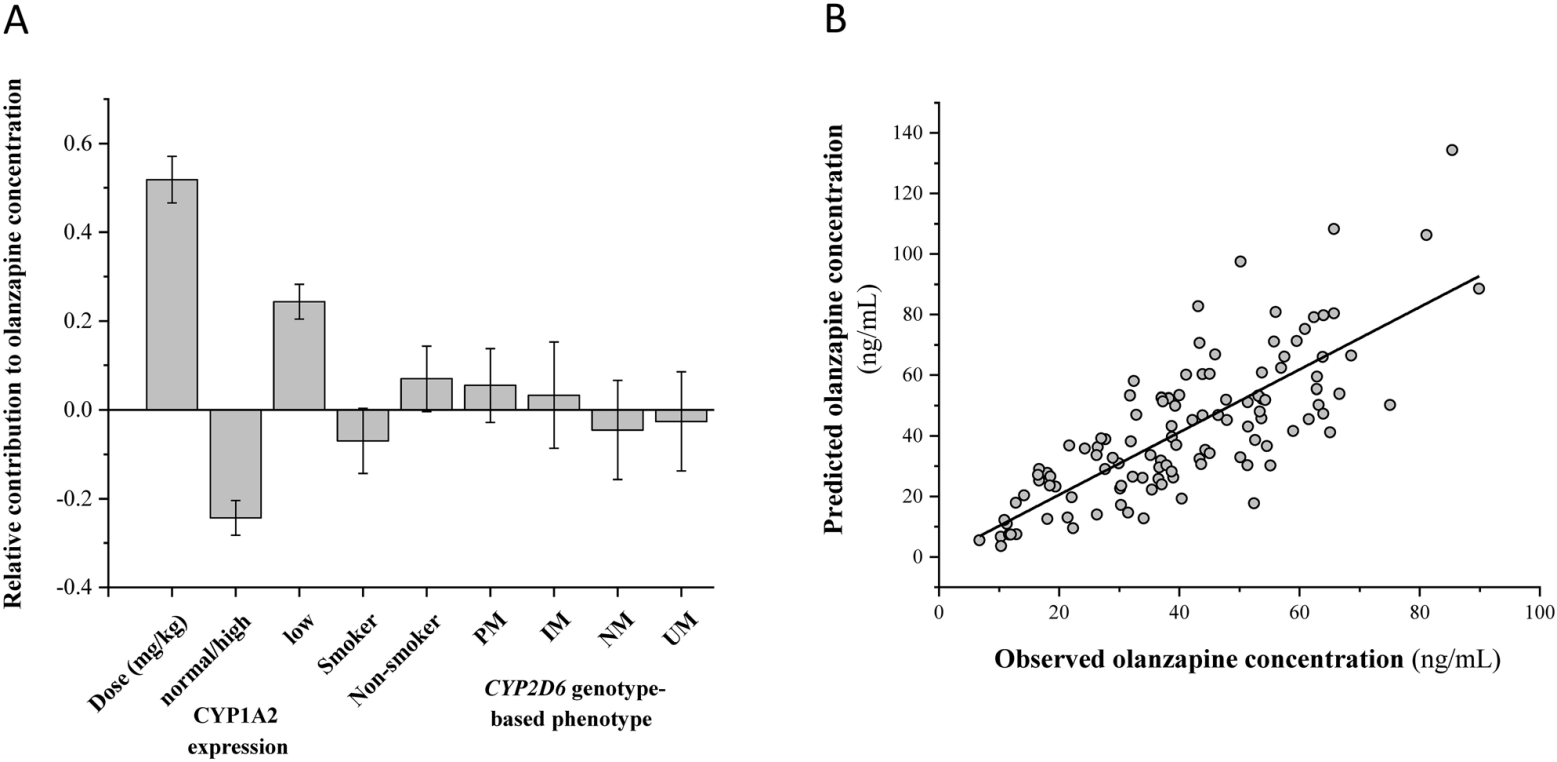
PLS model for olanzapine plasma concentration. (**A**) Principal component analysis with input variables of olanzapine daily dose/patients’ bodyweight, patients’ CYP1A2 expression, smoking behaviour and *CYP2D6* genotype-based phenotype; (**B**) Olanzapine plasma concentrations predicted by the PLS model.

In routine clinical practice, olanzapine is administered according to the consensus therapeutic guideline to achieve the optimal therapeutic concentration (20–80 ng/mL) for treatment of schizophrenia and bipolar disorder^14^ supplemented with monitoring the relief of symptoms and appearance of side effects. To determine the relative role of key factors influencing olanzapine plasma concentration in psychiatric patients, the PLS model was applied with olanzapine daily dose (mg/kg), *CYP1A2* genotype, CYP1A2 expression, *CYP2D6* genotype-based phenotype, sex, age and smoking behaviour as input variables and olanzapine plasma concentration as the output variable (Fig. 4A). The input variables without significant contribution to olanzapine concentration regarding the distribution of centered and scaled model coefficients, such as *CYP1A2* genotype, sex, age and *CYP2D6* genotype-based phenotype were eliminated from model building. The final PLS model equation with the main contributing input factors, such as olanzapine daily dose/bodyweight, CYP1A2 expression and smoking was described as follows:

$${\text{cc}}_{{{\text{olanzapine}}}} = \left( {3.187 + {\text{CYP1A2}} + 12.08 \times {\text{D}} + {\text{S}}} \right)^{2}$$

where “cc_olanzapine_„ is olanzapine plasma concentration predicted from the model (ng/mL), “CYP1A2” is − 0.989 for normal/high CYP1A2 expressers or 0.989 for low CYP1A2 expressers, “D” is the daily dose of olanzapine (mg/kg), “S” is − 0.273 for smokers or 0.273 for non-smoker patients. The value R^2^ (0.64) and Q^2^ (0.61) showed a considerable prediction power for the PLS model, suggesting that olanzapine daily dose, patient’s CYP1A2 expression phenotype and smoking were mostly responsible for olanzapine plasma concentration variability in psychiatric patients (Fig. 4B). It should be noted that the derived model is non-linear as best prediction was obtained for the squared root of the olanzapine plasma concentration.

## Discussion

High interindividual variability in olanzapine pharmacokinetics may impact both the patients’ response to the drug and the development of adverse effects which may eventually limit the success of antipsychotic therapy. The present study involving patients with psychiatric disorders investigated the role of *CYP1A2* and *CYP2D6* genetic variants and patients’ CYP1A2 metabolic capacity influenced by non-genetic factors (e.g., sex, age, smoking behaviour) in olanzapine exposure. Although CYP2D6 seems to have minor contribution to olanzapine metabolism and has negligible effect on plasma concentration *in vivo*^10,17,25,56^, interindividual variability in CYP1A2 activity has been reported to be associated with olanzapine exposure and patients’ dose requirement^50^.

In the patients of the present study, the trough concentrations of olanzapine substantially varied between 1.3 and 170 ng/mL (0.0042–0.54 µM), and at high plasma levels, oxidative enzymes other than CYP1A2 may be assumed to be additionally involved in olanzapine metabolism. However, one must also consider that the *Km* values (Michaelis constant) for the formation of olanzapine metabolites by various enzymes (14–22 µM for CYP1A2; 638 µM for CYP2D6; 228 µM for FMO3) far exceed the highest plasma concentrations in patients^57^; therefore, CYP1A2 was likely to be the dominant enzyme in the metabolism. Furthermore, the patients had a wide range of bodyweight from lean (43 kg) to obese (122 kg) that might have had an impact on olanzapine pharmacokinetics. The obesity is known to influence several physiological processes important in ADME (absorption, distribution, metabolism, excretion) parameters of drugs, e.g., increasing gut permeability and liver blood flow, altering drug distribution in tissues and drug metabolizing function of the liver^58^. Therefore, pharmacokinetic properties of a drug may be altered by obesity. Olanzapine is a lipophilic compound (logP is over 3) and may easily diffuse into adipose tissue increasing volume distribution^59^. However, CYP1A2 function has been reported to be unaltered in obese patients^60^, and the rate of olanzapine metabolism is not expected to be modified due to obesity.

The variability of CYP1A2 function is linked to genetic polymorphisms resulting in altered enzyme activity; however, haplotype misidentification from SNPs often leads to inconsistent phenotype estimation and allele frequency data in the literature^35,39,61–63^. Some clinical relevance has been attributed to CYP1A2*1F, one of the most frequently studied alleles^27^. Several authors considered CYP1A2*1F to be identical with − 163C > A SNP; however, − 163C > A appears to be in close genetic linkage with − 3860G > A, − 2467delT, − 739T > G or 2159G > A SNPs in CYP1A2*1J, CYP1A2*1K, CYP1A2*1L, CYP1A2*1M, CYP1A2*1V or CYP1A2*1W haplotypes^47,64^. Involving the most common SNPs in *CYP1A2* gene into the haplotype reconstruction, CYP1A2*1F was demonstrated to be one of the rarest *CYP1A2* alleles in the present patient population with a frequency of 0.4%, in contrast to the 32–57% of literature data^27^. The low prevalence thus did not allow to investigate the effect of CYP1A2*1F on enzyme activity and olanzapine exposure. Nevertheless, the contribution of − 163C > A to CYP1A2 inducibility has been well demonstrated, and several in vivo studies observed elevated CYP1A2 activity in − 163A carrier subjects with smoking or under CYP1A2 inducer omeprazole therapy^42,47,65,66^. In the present study, the smoker patients with − 163C/A or − 163A/A genotypes displayed significantly higher CYP1A2 mRNA expression than the − 163C/C carrier smokers; however, this higher inducibility of − 163A carriers was not manifested in low olanzapine plasma concentrations which was in line with the findings of Czerwensky et al.^23^. Decreased CYP1A2 activity was reported to be associated with − 3860G > A and to − 24667delT^37,38^; however, no significant association was found between these SNPs and olanzapine plasma concentrations in the patients involved in the present study. These results confirmed the observation of a recent clinical study that *CYP1A2* genotype has no significant predictive power for olanzapine exposure^67^. Similarly to *CYP1A2*, *CYP2D6* alleles had no effect on olanzapine exposure in psychiatric patients.

Strong correlation between hepatic CYP1A2 activity and mRNA expression has been demonstrated in previous studies with liver tissue donors^35,68^. Furthermore, CYP1A2 expression in leukocytes has been reported to reflect hepatic CYP1A2 activity; thus, leukocytes are considered to be appropriate biological samples for prediction of patients’ CYP1A2 metabolizing capacity^26^. In the patients with psychiatric disorders of the present study, significant association was observed between CYP1A2 expression in leukocytes and olanzapine metabolizing activity. The olanzapine concentration normalized by the dose/bodyweight was approximately twofold higher in the patients with poor CYP1A2 metabolizing capacity compared to intermediate/extensive metabolizer subjects. Several non-genetic intrinsic and environmental factors (e.g., sex, age, nutrition, diseases, hormonal status, smoking and medication) regulating *CYP1A2* transcription or inhibiting CYP1A2 enzyme function have been demonstrated to contribute to the interindividual variability of CYP1A2 phenotype^27,69,70^. Clear association between smoking and increased CYP1A2 activity has been described, and several components of tobacco smoke have been demonstrated to induce *CYP1A2* transcription via aromatic hydrocarbon receptor mediated pathway and to increase CYP1A2 metabolic capacity^71^. Furthermore, smoking, the well-studied behaviour seems to prevail within the population of psychiatric patients, primarily of those with schizophrenia^56,72,73^; therefore, an attention must be paid to the effect of smoking on olanzapine exposure. Approximately 1.5-fold higher olanzapine plasma concentrations were observed in the non-smoker patients of the present study than in smokers, which confirmed the findings of previous studies^17,50,74^. Low olanzapine exposure in smokers was attributed to the smoking induced increase in CYP1A2 expression and activity; therefore, patients’ smoking behaviour is suggested to be considered in optimal dose settings for efficient olanzapine therapy in psychiatric patients^75,76^. The PLS model built in the present study also supported the significant role of smoking and CYP1A2 expression as independent variables in olanzapine exposure. Considering these factors together, the model provided a good prediction for olanzapine serum concentrations.

Age has been suggested to have negative impact on drug metabolism, generally resulting in lower clearance in elderly patients (> 65) than in younger subjects^77^. An increase in olanzapine concentration by an average of 9.4% per decade of life was observed by Weiss et al.^78^. Sex differences also appear to influence olanzapine pharmacokinetics with reduced clearance in women that has been explained by the evidence for females having lower hepatic CYP1A2 activity^25,74,79^. However, in the present patient population, no statistically significant association was demonstrated between olanzapine exposure and age or sex, most probably due to other non-genetic variables influencing CYP1A2 function that were able to mask the effect of age and sex. Furthermore, it should be noted that only two patients were older than 65 years of age that did not allow to demonstrate a decrease in CYP1A2 function in elderly subjects.

Pharmacokinetic drug-interactions evoking CYP1A2 induction or inhibition of CYP1A2 activity can obviously modify the clearance of CYP1A2 substrate drugs^80,81^. It is highly relevant for psychiatric patients, because they are often under multidrug therapy including antipsychotics, mood stabilizers, antidepressant and sedative agents or under other drugs for treatment of comorbid medical conditions^82^. Co-administration of potent CYP1A2 inhibitor drugs (e.g. ciprofloxacin, fluvoxamine or oral contraceptives) has been reported to significantly elevate the serum concentrations of CYP1A2 substrates, while CYP1A2 inducer drugs, such as carbamazepine and omeprazole, are known to significantly increase the clearance of CYP1A2 substrates, including olanzapine^80,81,83^. In the present study, none of the patients were reported to receive fluvoxamine, carbamazepine or omeprazole, and only one subject displaying low CYP1A2 expression was on ciprofloxacin therapy; however, ciprofloxacin was likely to have negligible impact on the patient’s intrinsic poor metabolizer phenotype. No information about oral contraceptive therapy was available in the patients’ medication history which was one of the limitations of the present study.

Further limitation was that the effect of *CYP1A2* SNPs other than − 3860G > A (rs2069514), − 2467delT (rs35694136), − 739T > G (rs2069526), − 163C > A (rs762551) or 2159G > A (rs2472304) on olanzapine concentrations was not evaluated; however, other *CYP1A2* alleles associated with altered CYP1A2 activity or expression (e.g., CYP1A2*3, CYP1A2*4, CYP1A2*6, CYP1A2*7, CYP1A2*8, CYP1A2*11 and CYP1A2*15) do not occur or occur with low prevalence in Caucasian populations^49,63^. Second, due to low frequency of CYP1A2*1F allele (0.4%) in the patients involved in the present study, the effect of CYP1A2*1F on CYP1A2 function in olanzapine metabolism was not assessed.

## Conclusion

The present study focused on the impact of *CYP1A2* and *CYP2D6* genetic polymorphisms as well as CYP1A2 metabolizing capacity influenced by non-genetic factors (sex, age, smoking behaviour) on olanzapine blood concentration in patients with psychiatric disorders. CYP2D6 appeared to have negligible contribution to olanzapine metabolism, whereas a dominant role of CYP1A2 in olanzapine exposure was confirmed. However, we first demonstrated that CYP1A2 mRNA expression rather than *CYP1A2* genetic variability was associated with olanzapine concentration in patients. Involving the most common SNPs in *CYP1A2* gene into the haplotype reconstruction, CYP1A2*1F was found to be one of the rarest *CYP1A2* alleles in the present psychiatric population with a frequency of 0.4%, because the − 163C > A (rs762551) SNP designating CYP1A2*1F occurred in genetic linkage with other SNPs (− 3860G > A, − 2467delT, − 739T > G or 2159G > A) in CYP1A2*1L, CYP1A2*1M, CYP1A2*1V or CYP1A2*1W alleles. Significant contribution of − 163C > A to enhanced CYP1A2 inducibility was confirmed by an increase in CYP1A2 mRNA expression in smoker − 163A carriers. Tobacco smoking was proved to be a dominant non-genetic variable that increased olanzapine metabolizing capacity in patients, whereas sex and age appeared to have no impact on olanzapine exposure. Furthermore, it has been clearly demonstrated that patients’ olanzapine exposure (C/D) was in strong association with CYP1A2 mRNA expression; thus, assaying CYP1A2 mRNA level in patients’ leukocytes can be an appropriate tool for the estimation of their CYP1A2 metabolizing capacity and may facilitate to avoid misdosing induced adverse reactions or olanzapine inefficacy.

## Data Availability

The data that support the findings of this study have been deposited in the European Variation Archive (EVA)^84^ at EMBL-EBI under accession number PRJEB60477 (https://www.ebi.ac.uk/eva/?eva-study=PRJEB60477).

## Abbreviations

CPIC: Clinical Pharmacogenetics Implementation Consortium
CYP: Cytochrome P450
FMO: Flavin-containing monooxygenase
GAPDH: Glyceraldehyde 3-phosphate dehydrogenase
PCA: Principal component analysis
PCR: Polymerase chain reaction
PLS: Partial least-square
SNP: Single nucleotide polymorphism
UGT: Uridine diphosphate-glucuronyltransferase
